# A Scenario-Based Survey of Clinician Recommendations for Follow-Up Blood Cultures in Patients With Varying Risk of Persistent Gram-Negative Bacteremia

**DOI:** 10.1093/ofid/ofag562

**Published:** 2026-09-08

**Authors:** Roberta Monardo, Halie L Hotchkiss, Rebecca North, Olivia Lambert, Ilan S Schwartz, Marco Ripa, Vance G Fowler, Joshua T Thaden, Stacey A Maskarinec

**Affiliations:** Division of Infectious Diseases, IRCCS San Raffaele Scientific Institute, Milan, Italy; Division of Infectious Diseases, Duke University, Durham, North Carolina, USA; Center for the Study of Aging and Human Development, Duke University School of Medicine, Durham, North Carolina, USA; Center for the Study of Aging and Human Development, Duke University School of Medicine, Durham, North Carolina, USA; Division of Infectious Diseases, Duke University, Durham, North Carolina, USA; Division of Infectious Diseases, IRCCS San Raffaele Scientific Institute, Milan, Italy; Department of Medicine, Vita-Salute San Raffaele University, Milan, Italy; Division of Infectious Diseases, Duke University, Durham, North Carolina, USA; Duke Clinical Research Institute, Duke University, Durham, North Carolina, USA; Division of Infectious Diseases, Duke University, Durham, North Carolina, USA; Division of Infectious Diseases, Duke University, Durham, North Carolina, USA

**Keywords:** diagnostic stewardship, follow-up blood culture, gram-negative bacteremia, survey

## Abstract

**Background:**

The utility of follow-up blood cultures (FUBCs) in patients with gram-negative bacteremia (GNB) is unsettled. In practice, the use of FUBCs is variable. Understanding practice variation and drivers of FUBC ordering may clarify how clinicians identify patients at high risk for persistent GNB.

**Methods:**

We conducted a global survey (21 February 2024 to 9 April 2024) of healthcare workers using 10 hypothetical clinical scenarios to assess FUBC decision-making in GNB. Scenarios varied clinical factors and risk of persistence using the published AIMS scoring tool (*A*ntibiotics, *I*nfection source, *M*edical conditions, *S**erratia*). The anonymous web-based survey was distributed via listservs, email, and social media. Interrespondent agreement was assessed using Fleiss’ kappa (κ). Mixed-effect logistic regression identified factors associated with FUBC recommendations.

**Results:**

Among 864 clinicians from 50 countries, 691 (80%) provided recommendations across 10 scenarios (6910 recommendations). Most were physicians (86.5%) and infectious disease specialists (79.7%). Follow-up blood cultures were frequently recommended, with >50% of clinicians endorsing FUBCs in 6 of 10 scenarios. Overall agreement was fair (κ = 0.26; 95% confidence interval, .10–.43). In adjusted analyses, factors associated with FUBC recommendations included female respondent sex (adjusted odds ratio [aOR] 1.40; *P* = .042), higher AIMS score (aOR 1.78 per point; *P* < .0001), older patient age (aOR 2.90; *P* < .0001), inadequate source control (aOR 4.77; *P* < .0001), and clinical instability (aOR 114.99; *P* < .0001).

**Conclusions:**

In this survey study, clinicians frequently recommended FUBCs, particularly in clinical vignettes with higher AIMS scores, inadequate source control, and clinical instability. Only fair agreement across risk-stratified scenarios suggests substantial practice variation and supports the need for prospective studies to understand when and in whom FUBCs should be used.

## Teaser

This global survey examined clinician recommendations for follow-up blood cultures across patient scenarios of gram-negative bacteremia. While recommendations were more frequent in scenarios with a higher risk of persistent bacteremia, inadequate source control, and clinical instability, substantial practice variation highlights the need for prospective studies to determine when follow-up blood cultures are most useful.

Gram-negative bacteremia (GNB) is associated with high morbidity and mortality in hospitalized patients [1–3]. While follow-up blood cultures (FUBCs) are the standard of care for patients with *Staphylococcus aureus* bacteremia to help distinguish high-risk patients and guide treatment decisions, their role in GNB remains controversial [4–6]. Recent studies investigating the benefits of ordering FUBCs in all patients with GNB have yielded mixed results, yet they consistently demonstrate that persistent GNB is associated with a higher risk of death [5, 7, 8]. However, in the absence of guidelines, clinicians demonstrate considerable variability in ordering FUBCs for GNB management, but what influences these decisions is unknown [5, 7, 9].

To identify high-risk patients, our group developed the AIMS scoring instrument (*A*ntibiotics, *I*nfection source, *M*edical conditions, *Serratia*), which provides a numerical risk score (point range: 0–9) for persistent GNB in hospitalized adult patients [5]. Unlike other scoring tools that rely on variables requiring several days of hospitalization, the AIMS score is based on variables typically available at the time the index blood culture becomes positive to estimate the probability of persistent GNB [10, 11]. This timing more closely reflects when clinicians begin to risk-stratify patients for persistent bacteremia and make decisions regarding FUBCs. While not externally validated for predictive accuracy, the AIMS score was derived from a large cohort of prospectively enrolled hospitalized patients (>1700) with GNB over a 13-year period, providing a robust framework for evaluating clinician decision-making regarding the use of FUBCs [5].

The objective of this study is to characterize current FUBC practice patterns and identify factors influencing recommendations for FUBC ordering in patients with GNB. To accomplish this, we conducted a global survey examining FUBC ordering decisions using hypothetical clinical vignettes representing varying risk levels for persistent GNB as stratified by AIMS score. In addition, we examined respondent demographics and clinical variables not included in the AIMS score but previously associated with poor outcomes in GNB to identify factors that influence FUBC ordering recommendations [12, 13].

## METHODS

### Survey Instrument Development and Distribution

We developed an anonymous web-based survey to assess FUBC ordering recommendations, respondent demographics, and general practice patterns of clinicians when managing patients with bloodstream infection. Our survey uses previously validated instruments to assess decision-making, focusing on how clinicians choose to order additional diagnostic tests in patients with bacterial infections [14, 15]. Details regarding the survey components and the full survey instrument are included in the Supplementary Data.

### Ethical Approval

The study protocol and survey instrument were approved by the Duke Institutional Review Board. Respondents indicated their consent to participate by checking a mandatory box on the information page prior to the initiation of the survey.

### Data Collection and Management

The survey data were collected and managed using Qualtrics software tools hosted at Duke University. The survey link was distributed via email list to various national specialty societies, direct email contacts, and social media. Overall response rates or estimates could not be calculated because the survey link was anonymous, the survey link was posted on multiple social media platforms, and respondents were invited to forward the link to colleagues. To remove potential nontargeted respondents, records were screened for responses that consistently selected “other” for multiple-choice answers and for nonsensical responses provided in free-text fields [16]. The survey was made available from 21 February 2024, to 9 April 2024 (48 days) in the English language only.

### Statistical Analysis

Descriptive statistics were estimated to summarize the collected data. Data were represented as counts and percentages of the number of clinicians who answered the questions. Comparisons were evaluated by χ^2^ tests, with statistical significance determined at the α = .05 level. Interrater agreement across AIMS response categories was assessed using Fleiss’ kappa (κ) for nominal data. Mixed-effect logistic regression with random intercepts was used to determine factors associated with FUBC ordering recommendations. Independent variables found to exhibit a trend toward significance (*P* ≤ .15) in the univariate analysis were considered for inclusion in the model. Other candidate variables were selected based on clinical rationale for their potential relevance to the outcomes of interest. Data analyses were performed using SAS software version 9.4 (SAS Institute, Inc., Cary, NC, United States).

## RESULTS

**Figure 1.**
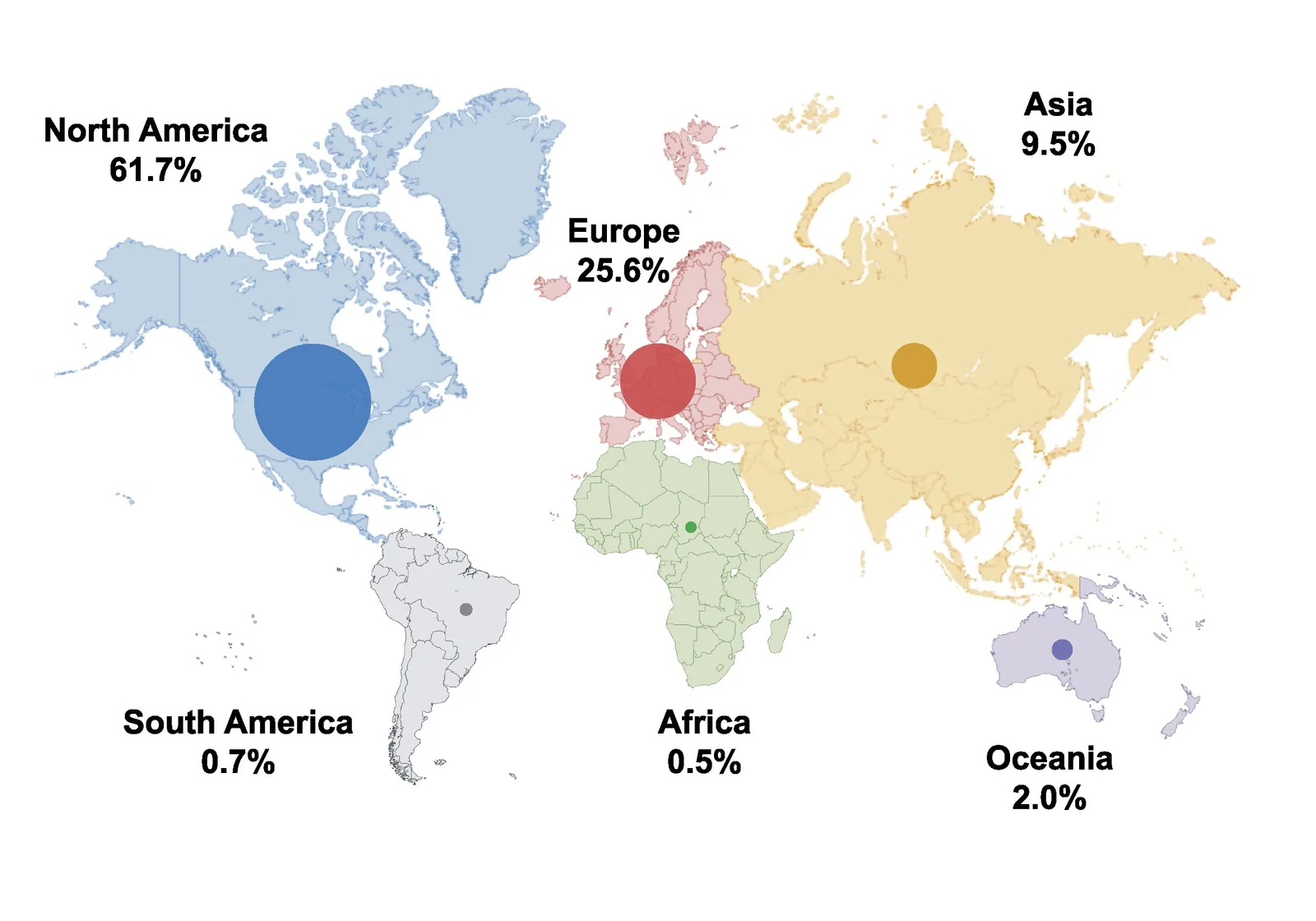
Global distribution of survey respondents (*n* = 864). Respondents per country: 50 unique countries over 6 continents participated, and participation ranged from 1 to 499 respondents per country. Created with BioRender.com.

**Figure 2.**
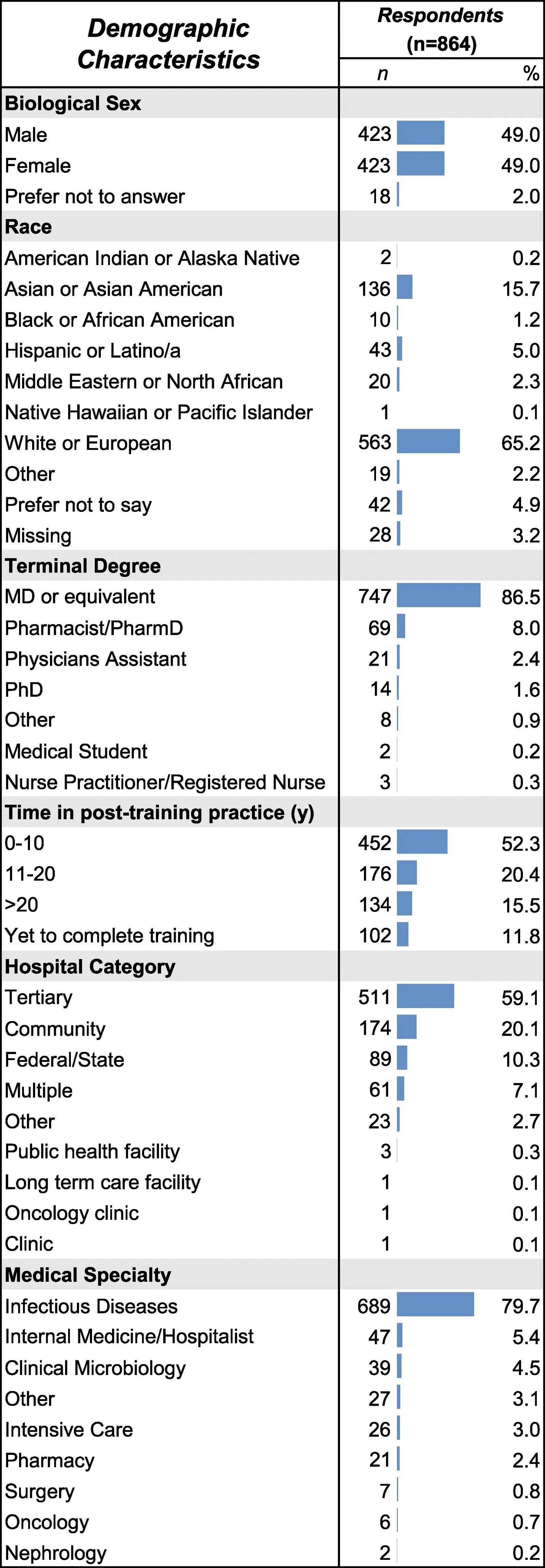
Demographic characteristics of survey respondents

A total of 864 individual survey responses were obtained. Of these, 691 clinicians completed all 3 sections of the survey (demographics, general practice questions, and hypothetical clinical vignettes), yielding an overall completion rate of 80% (691/864). The demographics section was completed by all clinicians (100% [864/864]), the general practice section by 92% (791/864), and the hypothetical vignette section by 80% (691/864).Across the 10 vignettes, clinicians provided a total of 6910 FUBC recommendations. No nontargeted responses were identified. The geographic distribution corresponded to 50 different countries on 6 continents: North America (533 responses, 61.7%), Europe (222 responses, 25.6%), Asia (82 responses, 9.5%), Oceania (17 responses, 2.0%), South America (6 responses, 0.7%), and Africa (4 responses, 0.5%) (Figure 1). Respondents were predominantly physicians (86.5%), most specializing in infectious diseases (ID) (79.7%), practicing at tertiary care hospitals (59.1%), and having completed training within the past 10 years (52.3%) (Figure 2). The most commonly reported primary practice focus was the care of immunocompetent patients (64%) (Supplementary Figure 1). Most clinicians reported managing 6–10 cases of bacteremia per month (22.8%) (Supplementary Figure 1).

### General Practice Patterns in the Management of Bacteremia

When managing patients with a known diagnosis of bacteremia (Gram-positive and Gram-negative), clinicians reported using several strategies, including evaluation for infection source (96.6%), response to antibiotic therapy (94.8%), assessment of antibiotic selection (88.2%), additional diagnostic imaging (69.8%), and FUBC acquisition (58.2%) (Supplementary Figure 1 and Table 1). When asked to identify clinical features indicative of an unfavorable clinical response, clinicians most commonly selected hypotension (77.6%) and fever (77.0%), followed by leukocytosis (50.4%), C-reactive protein elevation (CRP) (25.2%), and persistent bacteremia (4.9%) (Supplementary Figure 1 and Table 2). The majority of clinicians reported no institutional requirements or restrictions on ordering FUBCs for bacteremia due to any microbial species (91.2%).

### General and FUBC Practice Patterns Stratified by Geographic Location

The initial management of patients with a known diagnosis of bacteremia varied by geographic location. Clinicians in North America more frequently ordered FUBCs (62.7% vs 50.5%, *P* < .001) and diagnostic imaging (74.2% vs 62.4%, *P* < .001), compared with clinicians from other continents combined (Supplementary Table 3). Clinicians in North America more frequently identified leukocytosis (53.4% vs 45.4%, *P* = .029) and persistent bacteremia (6.5% vs 2.4%, *P* = .010) as indicators of unfavorable clinical response following 48 hours of empiric therapy (Supplementary Table 4). In contrast, clinicians outside North America more commonly relied on CRP elevation (47.8% vs 11.7%, *P* < .001) and procalcitonin levels (2.7% vs 0%, *P* < .001). Follow-up blood culture recommendation rates were similar between geographic regions in 7 of 10 hypothetical case scenarios describing patients with GNB (Supplementary Table 5). Additional descriptive analyses showing practice patterns and FUBC recommendations by continent are provided in Supplementary Tables 6–8; formal statistical comparisons were not performed because of the small sample sizes for several continents.

### General and FUBC Practice Patterns Stratified by Years in Clinical Practice

Clinicians were stratified by years in clinical practice to assess whether clinical experience influenced bacteremia management and FUBC ordering recommendations. No significant differences in the initial management of a patient with a known diagnosis of bacteremia were observed when clinicians were stratified by years in practice (Supplementary Table 9). Clinicians with more experience more frequently identified leukocytosis as an indicator of unfavorable response, whereas those yet to complete training more commonly relied on CRP elevation (Supplementary Table 10). Follow-up blood culture recommendation rates were similar across groups in 9 of the 10 hypothetical case scenarios describing patients with GNB (Supplementary Table 11).

### General and FUBC Practice Patterns Stratified by ID Versus Non-ID Specialists

The initial management of patients with a known diagnosis of bacteremia varied by whether the clinician was an ID specialist. Infectious disease specialists more frequently indicated that they would examine for infection source (98.3% vs 89.3%, *P* < .001), order diagnostic imaging (72.1% vs 59.7%, *P* = .003), and monitor response to antibiotic therapy (96.1% vs 89.3%, *P* < .001) (Supplementary Table 12). Non-ID specialists more commonly relied on CRP elevation (34.9% vs 22.9%, *P* = .002) to assess whether a patient had a favorable response to clinical management (Supplementary Table 13). Follow-up blood culture rates were similar between ID and non-ID specialists in 7 of 10 hypothetical case scenarios describing patients with GNB (Supplementary Table 14).

### General and FUBC Practice Patterns Stratified by Hospital Category

Clinicians were stratified by hospital category to assess whether practice setting influenced bacteremia management and FUBC ordering recommendations. Examining for infection source and monitoring response to antibiotic therapy were most frequently reported by clinicians practicing in federal or state hospitals (Supplementary Table 15). Leukocytosis was most frequently identified as an indicator of an unfavorable response by clinicians practicing in community hospitals, whereas procalcitonin and lactate elevations were most frequently identified by those practicing across multiple hospital settings (Supplementary Table 16). Follow-up blood culture rates did not differ significantly across hospital categories in any of the 10 hypothetical case scenarios describing patients with GNB (Supplementary Table 17).

### Practice Patterns and Rationale for FUBC Use in GNB

**Figure 3.**
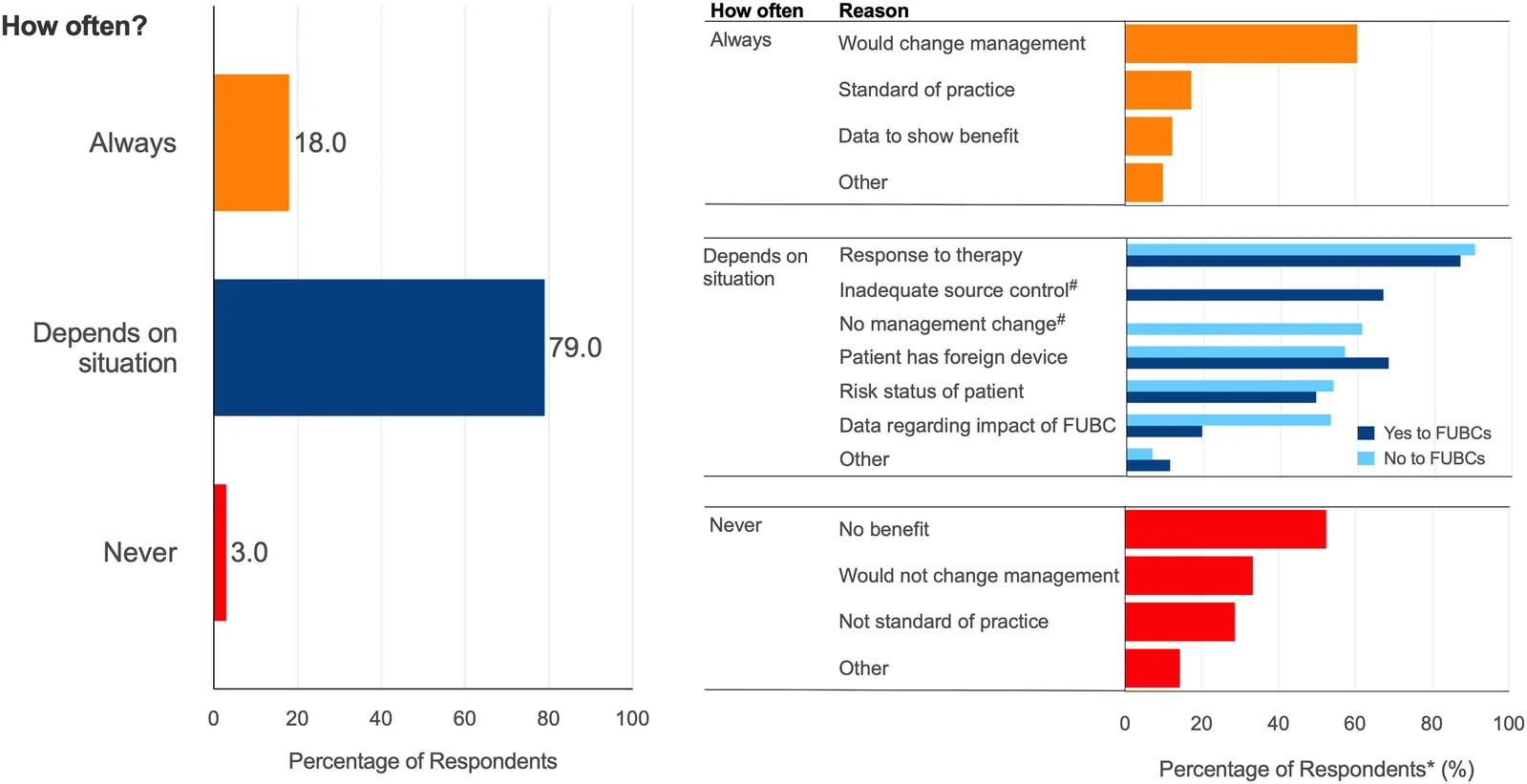
Clinician practices and rationale for obtaining follow-up blood cultures (FUBCs) in gram-negative bacteremia (GNB) (*n* = 791). (Left) Self-reported frequency of FUBCs for patients with GNB in routine clinical practice, categorized as “Always,” “Depends on the situation,” or “Never.” (Right) Reasons cited by clinicians for their selected practice patterns. #Indicates survey response options that were displayed only when clinicians selected a specific conditional response (Yes or No). Percentages may exceed 100% because respondents were allowed to select multiple responses (*).

Practice patterns for FUBCs ordering in GNB varied among clinicians. Overall, 18% reported routinely obtaining FUBCs in all cases, 79% reported selective ordering based on clinical circumstances, and 3% reported never obtaining FUBCs (Figure 3). Clinicians who reported “always” obtaining FUBCs most commonly cited their use to guide management decisions (60.5%). Among those who selected “depends on situation,” decisions on FUBC ordering were influenced by a range of different factors, including response to therapy, adequacy of source control, and presence of intravascular devices. Notably, responses regarding the impact of FUBCs in patients with GNB, based on published data, demonstrated the greatest variability (19%–53%). Clinicians who reported never obtaining FUBCs most often cited a lack of clinical benefit (52.4%), no anticipated change in management (33.3%), or deviation from their usual clinical practice (28.6%).

### FUBC Recommendations Across Hypothetical Clinical Scenarios Stratified by AIMS Score

**Figure 4.**
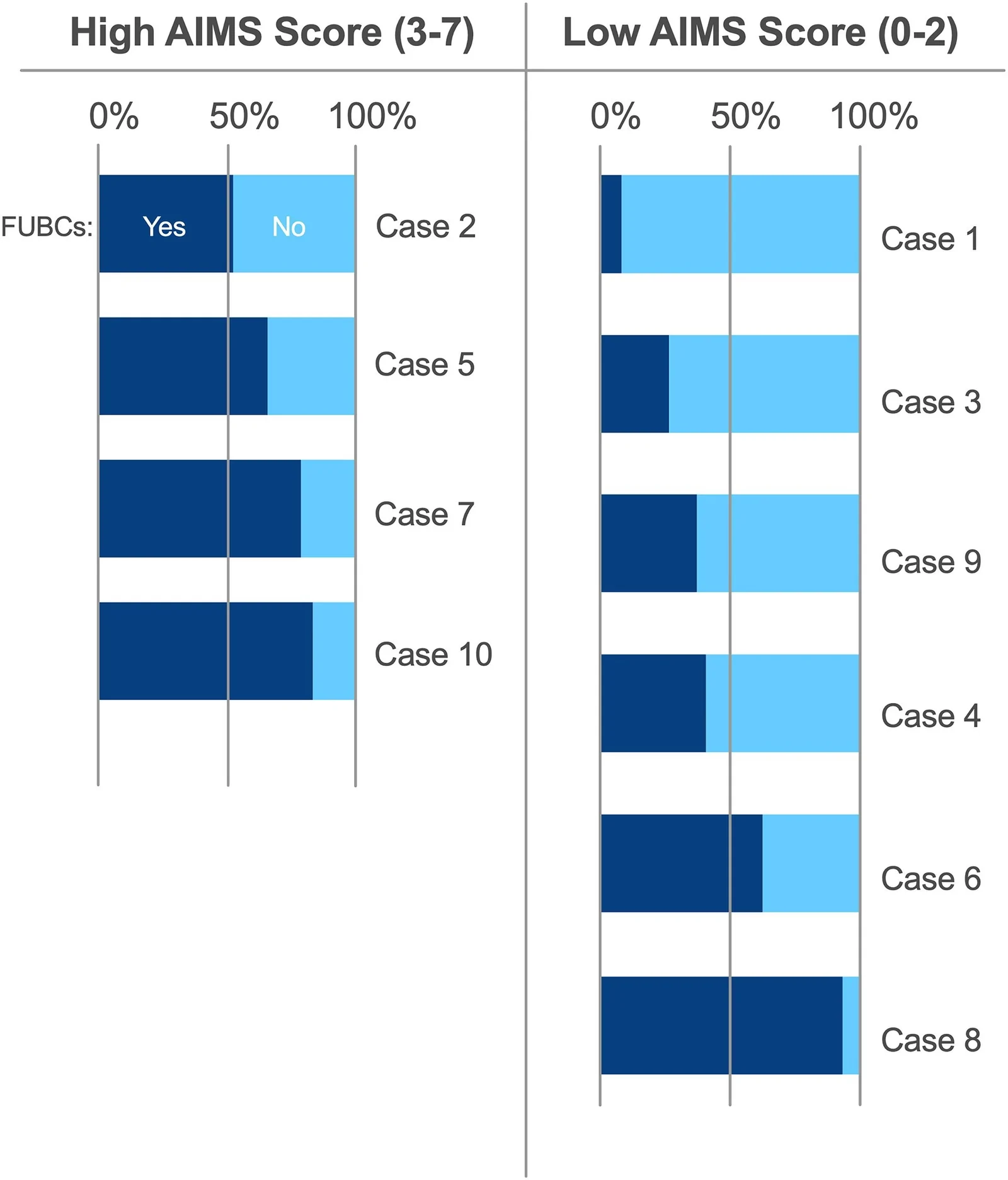
Survey responses from clinicians (*n* = 691) indicating whether they would obtain follow-up blood cultures (FUBCs) for hypothetical patient cases of gram-negative bacteremia (GNB) stratified by AIMS score (*A*ntibiotics, *I*nfection source, *M*edical conditions, *Serratia*). Cases were grouped into High AIMS Score (3–7) and Low AIMS Score (0–2) categories. Each horizontal bar represents the proportion of clinicians who selected “Yes” (dark blue; left side of bar) or “No” (light blue; right side of bar) for obtaining FUBCs in a given case.

Follow-up blood cultures were frequently recommended, with more than 50% of all clinicians recommending FUBCs in 6 of 10 scenarios (Figure 4). Overall, there was fair agreement among clinicians regarding FUBC ordering when evaluating all 10 hypothetical clinical scenarios (Fleiss’ κ = 0.26; 95% confidence interval [CI], .10–.43). Variation in FUBC ordering recommendations and interrespondent agreement were observed when scenarios were grouped by AIMS score. Although scenarios with an AIMS score ≥3 (corresponding to an approximate risk of persistent bacteremia of ∼20%) [5] were associated with higher rates of FUBC recommendations, responses among clinicians were more evenly divided in these cases, resulting in lower interrater agreement (Fleiss’ κ = 0.07; 95% CI, .02–.11). In contrast, lower-risk scenarios (AIMS score 0–2) demonstrated a wide range of responses, with FUBC recommendations ranging from 8% to 93%. Interrater agreement was slightly higher in these lower-risk scenarios (Fleiss’ κ = 0.30; 95% CI, .03–.56), suggesting somewhat greater consistency among clinicians in their recommendations within these cases.

As an exploratory analysis, we evaluated FUBC recommendations for each vignette among clinicians who reported that they “always,” “sometimes,” or “never” order FUBCs in patients with GNB (Supplementary Table 18). A notable discrepancy was observed among clinicians who reported that they “never” order FUBCs (n = 18). Among this group of clinicians, FUBCs were recommended in 9 of 10 vignettes, including 89% (16/18) of clinicians recommending FUBCs for Case 8.

### Factors Associated With FUBC Recommendations Across Hypothetical Case Scenarios

**Figure 5.**
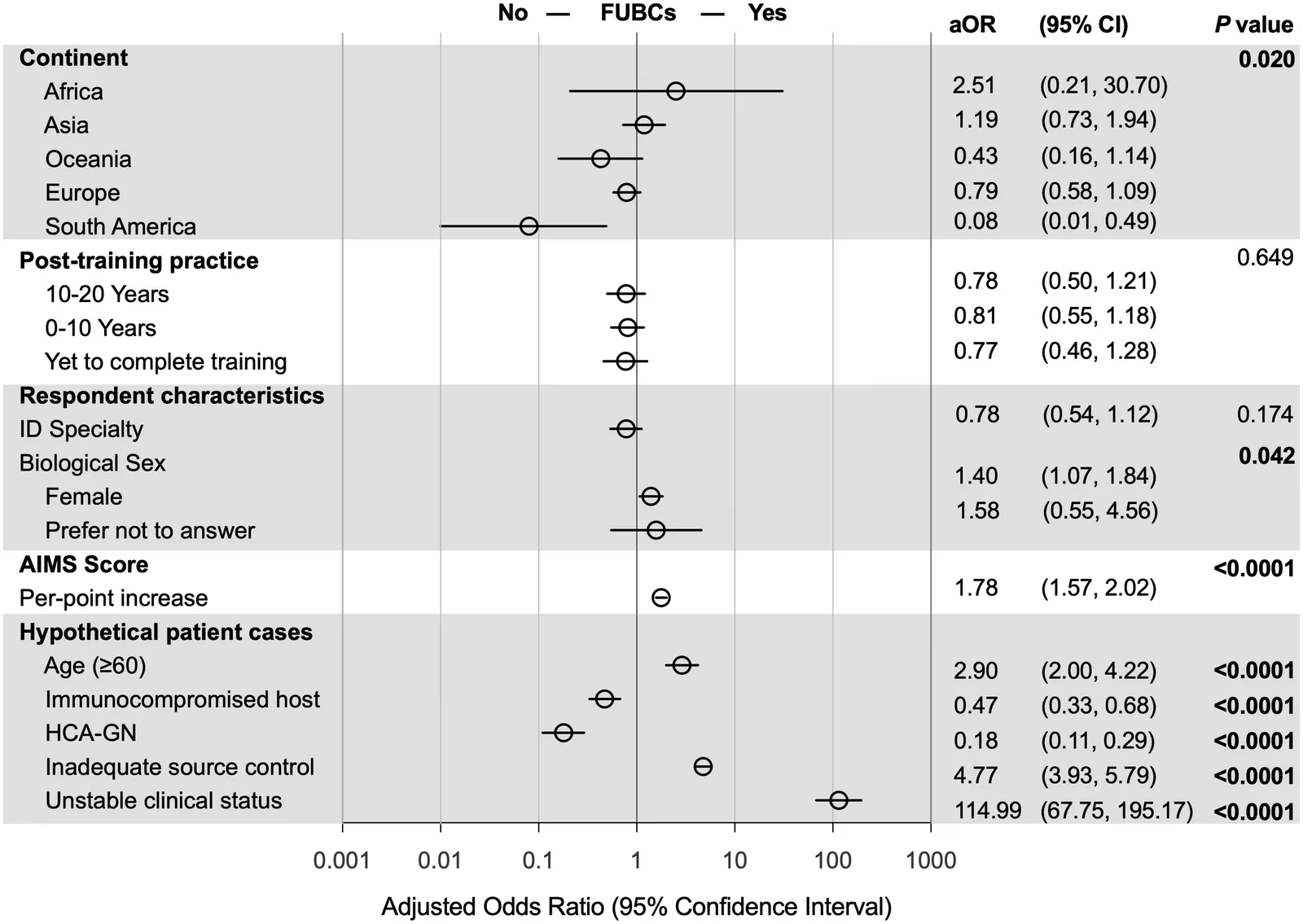
Factors associated with recommending follow-up blood cultures (FUBCs) in hypothetical cases of patients with gram-negative bacteremia (GNB). Adjusted odds ratios were estimated using multivariable mixed-effects logistic regression with a respondent-level random intercept. Reference categories were North America for continent, >20 y for posttraining practice, noninfectious disease (ID) specialist for ID specialty, male for biological sex, age <60 y, immunocompetent host, non-healthcare-associated gram-negative (HCA-GN) organisms for the HCA-GN variable, adequate source control, and stable clinical status. The estimate for the AIMS score represents the adjusted odds ratio associated with a 1-point increase in score. *P* values ≤.05 were considered statistically significant and are shown in bold.

A multivariable mixed-effects logistic regression model with a respondent-level random intercept was used to identify factors associated with recommending FUBCs across hypothetical clinical scenarios of GNB while accounting for repeated responses from the same respondent (Figure 5). Compared with clinicians who identified as male, those who identified as female were more likely to recommend FUBCs across all case scenarios (adjusted odds ratio [aOR] 1.40; 95% CI, 1.07–1.84). Higher AIMS scores were also associated with increased recommendations for FUBCs (aOR 1.78 per point increase; 95% CI, 1.57–2.02). Clinical features of the hypothetical patient that were associated with a greater likelihood of recommending FUBCs included age ≥60 years (aOR 2.90; 95% CI, 2.00–4.22), inadequate source control (aOR 4.77; 95% CI, 3.93–5.79), and clinical instability after 48 hours of antibiotic therapy (aOR 114.99; 95% CI, 67.75–195.17). In contrast, compared with clinicians from North America, respondents from South America were less likely to recommend FUBCs across scenarios (aOR 0.08; 95% CI, .01–.49). No significant differences in FUBC recommendations were observed among clinicians from other continents compared with those from North America. Additional factors associated with a lower likelihood of recommending FUBCs included immunocompromised status of the hypothetical patient (aOR 0.47; 95% CI, .33–.68) and healthcare-associated gram-negative organisms (aOR 0.18; 95% CI, .11–.29).

## DISCUSSION

Our global survey study revealed 3 key observations around practice patterns and factors influencing FUBC ordering in patients with GNB. First, clinicians commonly recommend FUBCs in patients with GNB. Second, substantial practice variation exists regarding when FUBCs should be obtained. Finally, clinicians appear to actively risk-stratify patients when deciding whether to obtain FUBCs, incorporating clinical indicators of infection severity and the likelihood of persistent bacteremia as reflected by the AIMS score. In the context of an increasingly common yet potentially lethal infection, the combination of frequent FUBC ordering and only fair interrater agreement highlights the need for prospective studies to define optimal strategies for FUBC use in GNB [17].

Our survey study demonstrated that clinicians frequently recommend FUBCs in the management of GNB. Nearly 1 in 5 respondents reported routinely obtaining FUBCs in all cases of GNB, while approximately 4 in 5 reported obtaining FUBCs selectively based on individual patient scenarios. These rates are largely consistent with cohort studies demonstrating that FUBCs are commonly obtained in hospitalized patients with GNB [5, 7, 9]. Consistent with these findings, hypothetical clinical vignettes elicited high rates of FUBC recommendations across the majority of cases. Prior studies have demonstrated geographic and ID subspecialty-related differences in bacteremia management [15, 16, 18, 19]. We observed similar differences in practice patterns in our study and further examined whether FUBC recommendations varied across these groups. Although modest differences in ordering rates were observed for individual vignettes, overall use remained high across geographic regions, years in practice, and ID subspecialization. While we did not directly assess the contextual or infrastructure-related factors that might explain regional variation in FUBC use, prior studies have shown that blood culture utilization varies across hospital settings and may be influenced by supply availability, specimen transport, staffing, and processing costs, particularly in resource-limited settings [20–23]. Statistically significant regional differences in imaging and inflammatory biomarker use may likewise reflect differences in resource availability or local practice. In the adjusted model, geographic differences were limited, and neither ID specialty nor years in practice was associated with FUBC ordering. Taken together, these findings indicate that FUBC ordering is a broadly shared practice and suggest that clinicians continue to perceive value in obtaining FUBCs despite the absence of professional society guidelines regarding their use and mixed evidence from cohort studies regarding their clinical impact.

Although respondents frequently recommended FUBCs in cases of GNB, only fair agreement was observed regarding when repeat cultures should be obtained, suggesting limited consensus on which clinical scenarios warrant FUBCs. This finding also highlights that despite increased focus on FUBC use in GNB, considerable variability in ordering practices remains even among ID specialists, who comprised the majority of respondents and are frequently consulted in the management of complicated bloodstream infections [24–26]. Surprisingly, disagreement was most notable in scenarios with a high AIMS score, which is associated with an increased risk of persistent GNB [5]. Higher-risk clinical scenarios would typically be expected to produce stronger agreement regarding diagnostic testing. Instead, although FUBC recommendations increased in all high-risk cases, responses were more evenly divided, suggesting that risk designation does not necessarily translate into consensus regarding clinical decision-making. For example, a global survey study recently demonstrated substantial variability in the use of 18F-fluoro-deoxyglucose positron emission tomography/computed tomography (18F-FDG PET/CT) for persistent *S. aureus* bacteremia, even in highly resourced countries, despite the well-recognized prognostic significance of this complication [16]. Practice variation in diagnostic testing strategies for the management of bacteremia has also been noted in multiple studies evaluating echocardiography use in patients with *S. aureus* bacteremia [15, 16, 27, 28]. In a similar survey using hypothetical clinical vignettes stratified by infective endocarditis risk according to the VIRSTA score, Heriot et al [15] likewise observed substantial disagreement among clinicians regarding echocardiography strategies across all scenarios of *S. aureus* bacteremia, particularly in those designated as low risk. To our knowledge, this is the first study to survey clinicians regarding FUBC ordering practices in GNB, limiting our ability to determine whether these patterns of agreement and practice variation are generalizable to other populations.

One potential explanation for the variation in FUBC recommendations among higher-risk vignettes is that clinicians may have interpreted the clinical implications of these cases differently. Despite sharing a higher-risk designation, the cases varied in trajectory, infection source, causative organism, and source control status. Clinicians may weigh some components more heavily than others and incorporate factors beyond the score, particularly clinical stability and source control status. These patterns suggest that the broader clinical context may shape how clinicians interpret the estimated risk of persistent bacteremia to determine whether to recommend FUBCs. Although this study could not determine the specific factors underlying disagreement, complementary methods such as discrete choice experiments, factorial vignette studies, and mixed-methods implementation research could quantify how clinicians weigh individual and combined risk factors across clinical contexts. In addition to clinical trials, these approaches could inform risk-stratified diagnostic stewardship interventions and guidance on FUBC use.

Standardized hypothetical clinical scenarios enabled the identification of respondent and patient characteristics associated with FUBC ordering recommendations. Patient-related variables reflecting infection severity, including source control and clinical status, showed the strongest associations with FUBC ordering recommendations. Case 8 illustrates the influence of clinical status, as nearly all respondents recommended FUBCs for a recent kidney transplant recipient with *Stenotrophomonas maltophilia* bacteremia who remained critically ill after 48 hours of therapy. While the organism and recent transplantation may have contributed, clinical instability was likely an important driver of FUBC recommendations. These findings are consistent with real-world practice, as FUBCs are often used to evaluate for complicated infection (eg, uncontrolled source or metastatic infection) and to inform subsequent therapeutic strategies [5, 29]. Moreover, these factors align with clinicians’ most frequent selections when describing their general management approach to patients with bacteremia, namely examining for infection source and monitoring patient response to antibiotic therapy.

Although diagnostic stewardship studies specifically addressing FUBCs in GNB remain limited, available evidence supports selective, risk-based rather than routine collection [30]. Our findings reinforce the need for this approach: FUBCs were frequently recommended across scenarios, with limited agreement regarding when to obtain them, although clinical instability and select clinical circumstances influenced ordering. Together, these findings support clearer guidance to target FUBCs to patients most likely to benefit.

Previously identified host and microbial risk factors for poor outcomes in GNB, including immunocompromised host status and bacteremia due to healthcare-associated organisms, were not associated with increased FUBC ordering recommendations in case scenarios. Several explanations may account for this finding. First, clinicians may prioritize risk factors for persistent bacteremia, such as endovascular infection or inadequate source control, rather than microbial characteristics associated with mortality when deciding whether to obtain FUBCs. Second, clinicians may apply a more contemporary and nuanced definition of the immunocompromised host than the simplified definition used in the regression model, potentially contributing to variation in FUBC ordering recommendations. Support for this explanation is derived from a recent study examining GNB management practices among major immunocompromised populations, including solid organ transplant, hematologic malignancy, and metastatic solid cancer. Follow-up blood cultures were obtained more frequently in solid organ transplant and hematologic malignancy populations, and FUBC acquisition was associated with lower mortality in the overall cohort, although its impact was uncertain in the solid organ transplant subgroup and not observed in patients with hematologic malignancy [31]. These findings suggest that clinicians may interpret immunocompromised status heterogeneously, thereby influencing how this variable impacts FUBC ordering.

Another notable finding was the association between the clinician's self-identified sex and FUBC recommendations. This observation is consistent with emerging literature suggesting physician sex–related differences in clinical decision-making, with studies reporting that female physicians may perceive clinical risk more highly and therefore request additional diagnostic testing or referrals compared with their male counterparts [32–36].

## LIMITATIONS

Our study has some limitations related to the design, dissemination, and completion of the survey. Our survey did not contain a trackable URL; therefore, a response rate could not be determined. Clinicians who completed the survey were largely from North America and Europe and thus do not fully represent the global community of healthcare professionals. In addition, the survey was administered only in English, which may have further limited participation from certain regions. Most of our survey respondents were ID specialists, which may limit external generalizability to the practice of non-ID specialists or under different diagnostic stewardship constraints. In particular, hospitalists and intensivists, who frequently make FUBC decisions, were underrepresented. Furthermore, specialists who subspecialized in infections in immunocompromised hosts comprised a relatively large number of respondents, and their practices may not reflect those of general ID specialists. It is possible that the responses of ID specialists who primarily care for immunocompromised hosts could have been influenced by their typical practice, even in hypothetical scenarios that involved patients who were not immunocompromised. Moreover, our survey distribution mechanisms were limited, making the assessment of representativeness uncertain.

Our survey was limited in that it only presented 10 hypothetical scenarios, all of which are simplifications of complicated disease processes and patient medical histories and cannot fully capture real-world clinical decision-making. Only 2 scenarios clearly involved immunocompromised patients and included other case-specific characteristics, warranting cautious interpretation of the unexpected association between immunocompromised status and lower FUBC recommendations. Additionally, the vignettes were constructed to represent a range of AIMS scores, which may have introduced bias toward the component variables. It is important to restate that, while the AIMS score is derived from a large prospective observational cohort study, it has not been externally validated. This limits interpretation of the observed relationship between AIMS score and FUBC recommendations, including the attenuated association among respondents outside North America. This survey captures stated recommendations to hypothetical vignettes, not actual bedside ordering behavior. Responses may also have been influenced by social desirability or demand characteristics if respondents perceived that recommending FUBCs was expected, potentially resulting in overestimation of their use. Thus, these results are best interpreted as a study of clinical decision-making rather than a direct assessment of utilization.

## CONCLUSION

This survey suggests that clinicians frequently obtain FUBCs in patients with GNB. However, substantial disagreement among clinicians regarding when FUBCs should be obtained highlights ongoing variability in clinical practice and reinforces the need for clinical trials and complementary studies of clinician decision-making to define optimal strategies for FUBC use. Risk-based implementation strategies that prioritize FUBCs in high-risk patients while minimizing unnecessary testing in low-risk patients represent an opportunity to improve outcomes while advancing diagnostic stewardship.

## Supplementary Material

ofag562_Supplementary_Data

## Supplementary Data

Supplementary materials are available at *Open Forum Infectious Diseases* online. Consisting of data provided by the authors to benefit the reader, the posted materials are not copyedited and are the sole responsibility of the authors, so questions or comments should be addressed to the corresponding author.
