# Supplementary material for: A Scenario-Based Survey of Clinician Recommendations for Follow-Up Blood Cultures in Patients With Varying Risk of Persistent Gram-Negative Bacteremia
Source: Open Forum Infect Dis. 2026 Sep 8;13(9):ofag562. doi: 10.1093/ofid/ofag562 (PMC13601797; doi:10.1093/ofid/ofag562)
Supplement: Supplementary file 1 [file ofag562_supplementary_data.docx]

**SUPPLEMENTAL MATERIALS**

**Survey structure**

**Hypothetical clinical scenarios were generated with varying patient age, host immunosuppression, clinical status after 48 hours of antibiotics, concern for inadequate source control, microbial species characteristics, and risk of persistent gram-negative bacteremia (GNB) based on our published AIMS scoring tool.^1^** Each respondent/clinician was given the same 10 hypothetical scenarios. For each case scenario, clinicians were asked to select whether or not they would recommend ordering follow-up blood cultures (FUBCs). Clinicians were not informed that the scenarios were based on a persistent GNB scoring tool or that the scenarios were generated specifically to vary their risk of persistent GNB.

Clinicians were asked to provide non-identifying demographic information, including country of practice, terminal degree, medical specialty, location of practice, focus of practice, hospital category, years of post-training experience, and monthly GNB caseload.

Clinicians were also asked about their approach to managing patients with a known diagnosis of bacteremia. Specifically, respondents were asked to identify clinical factors that indicate an unfavorable response to empiric therapy, how their management practices differ based on whether the bacteremia is caused by gram-positive or gram-negative organisms, general ordering practices, and motivations for obtaining FUBCs in patients with GNB, and whether or not some institutional policies or guidelines restrict or mandate FUBC ordering in GNB.

**Definitions**

AIMS Score Point Allocation for Persistent Gram-Negative Bacteria

| **Variable** | **Point Allocation** |
| --- | --- |
| **Antibiotics** |  |
| days to effective therapy (≥1) | **1** |
| **Infection source** |  |
| endovascular | **3** |
| gastrointestinal | **1** |
| genitourinary | **1** |
| other | **3** |
| **Medical co-morbidities** |  |
| cardiac device present | **1** |
| corticosteroid use | **1** |
| hemodialysis dependence | **1** |
| **Species** |  |
| *Serratia* species | **2** |
| **AIMS RISK SCORE** | **TOTAL (0-9)** |

***Classification of microbial groups***

Gram-negative organisms were categorized into two groups for analytic purposes, based on typical ecological niche and reported associations with healthcare exposure.^2-4^ These groupings do not represent strict taxonomic classifications.

Healthcare-associated gram-negative **(HCA-GN)** organisms were defined as gram-negative bacteria that are not predominant components of the normal gastrointestinal microbiota and are commonly associated with environmental reservoirs and healthcare settings. This group included *Pseudomonas aeruginosa, Stenotrophomonas maltophilia, and Serratia marcescens*.

Gut-derived gram-negative organisms were defined as gram-negative bacteria that commonly colonize the human gastrointestinal tract and are frequently associated with infections originating from the gastrointestinal or urinary tract. This group included *Escherichia coli, Klebsiella pneumoniae, Proteus mirabilis, Morganella morganii, and Bacteroides fragilis*. The reference group used in the regression analysis included all other pathogens represented in the clinical vignettes.

**Immunocompetent** was defined based on a previous definition^5^, as an [absence](https://www.sciencedirect.com/topics/pharmacology-toxicology-and-pharmaceutical-science/epileptic-absence) of any of the following comorbidities: (a) diabetes mellitus, (b) human immunodeficiency virus (HIV), (c) [hemodialysis](https://www.sciencedirect.com/topics/pharmacology-toxicology-and-pharmaceutical-science/hemodialysis) dependence, (d) active [malignancy](https://www.sciencedirect.com/topics/medicine-and-dentistry/cancer), and (e) receipt of [immunosuppressive](https://www.sciencedirect.com/topics/medicine-and-dentistry/immunosuppressive-drug) medications. Also included in this definition for this survey study included the absence of the following conditions: advanced/end-stage liver disease or cardiomyopathy.

***Advanced patient age*** was defined as greater than or equal to 60 years of age.

A hypothetical patient in a case scenario was considered “**clinically stable**” if the vignette included the following statement: “The patient initially demonstrated features of severe sepsis but has since stabilized.” (Cases 1-7, 9). Otherwise, it was considered “**clinically unstable**” (Cases 8, 10).

Cases designated as having potential concerns for “**inadequate source control**” contained phrases such as: “abscess that is not amenable to drainage;” and described the presence of infections that may involve invasive/implanted devices such as “ventricular peritoneal shunt infection” (Cases 2, 5, 6, 7, 10).

**Pilot testing**

The survey was piloted among 11 physicians (Italy, USA) who provided feedback on the number, structure, and content of the survey questions and the appropriateness of the demographic questions.

**Survey Instrument**

**1.** Thank you for your participation in this survey. This survey is voluntary, and no protected health information (PHI) will be collected. This survey is considered research and has been approved by the Duke University Institutional Review Board (Protocol ID: Pro00114863; PI: Stacey Maskarinec, MD, PhD).

[ ] I agree to participate

**2. In what country do you practice?**

Dropdown list of countries for selection

**3. What is your terminal degree?**

MD

DO

MD, PhD

MBBS

PhD

NP

PA

RN

Other, specify

**4. What is your primary area of medical specialty?**

Infectious disease

Clinical microbiology

Internal medicine/Hospitalist

Surgery

Nephrology

Oncology

Intensive Care Unit

Other, specify

**5. Please select the hospital category most appropriate for your practice:**

Tertiary hospital

Community hospital

Federal/state hospital

Multiple hospital types

Other, specify

**6. What is the primary focus of your practice?**

Immunocompetent patients

HIV/AIDS

Transplant/immunocompromised host

Other, specify

**7. How many years have you been in post training practice?**

Yet to complete my training

0-10 years

11-20 years

more than 21 years

**8. My race or ethnicity is best described as: (Please select all that apply)**

- American Indian or Alaska Native (e.g., Navajo Nation, Blackfeet Tribe, Inupiat Traditional Gov't., etc.)
- Asian or Asian American (e.g., Chinese, Japanese, Filipino, Korean, South Asian, Vietnamese, etc.)
- Black or African American (e.g., Jamaican, Nigerian, Haitian, Ethiopian, etc.)
- Hispanic or Latino/a (e.g., Puerto Rican, Mexican, Cuban, Salvadoran, Colombian, etc.)
- Middle Eastern or North African (e.g., Lebanese, Iranian, Egyptian, Moroccan, Israeli, Palestinian, etc.)
- Native Hawai`ian or Pacific Islander (e.g., Samoan, Guamanian, Chamorro, Tongan, etc.)
- White or European (e.g., German, Irish, English, Italian, Polish, French, etc.)
- My race or ethnicity is best described as:  (Feel free to use the text box and/or you can simply select categories above.)
- Prefer not to say

**9. Please select your sex**

Male

Female

Transgender female

Transgender male

Nonbinary

Prefer not to answer

**10. How do you approach a patient with a known diagnosis of bacteremia? (Please select all that apply)**

Examine patient to identify source

Order additional blood cultures to document clearance

Monitor patient response to antibiotic therapy

Order diagnostic imaging

Remove indwelling lines

Assess antibiotic choice based on institutional susceptibility data

Other

**11. What determines an unfavorable response in bacteremic patients following empiric therapy (48 hours)? Please select all that apply**

Fever

no change/increase leucocytosis

no change/increase of C-reactive protein

Hypotension

Other

**12. Is your clinical approach different if bacteremia is due to a gram-positive or a gram-negative organism?**

No, my approach is the same

Yes, my approach is different

**13. Approximately how many cases of gram-negative bacteremia do you see each month?**

0-2cases

3-5 cases

6-10 cases

11-20 cases

>20 cases

**14. Are there any institutional policies or guidelines that RESTRICT you from ordering follow up blood cultures in patients with gram-negative bacteremia?**

No

Yes

**15. In your clinical practice, how often do you order follow-up blood cultures in patients with Gram-negative bloodstream infection?**

- Never
- Only in certain cases
- Always

If responded “Negative” – directed to this question:

Why do you never order follow-up blood cultures in patients with Gram-negative bloodstream infection?

Would not change management based on culture results

Not standard of practice in affiliated hospital

No data to suggest benefit of follow up blood cultures

Other

If responded “Only in certain cases” – directed to these 2 questions:

*In what scenarios do you choose to order follow-up blood cultures in patients with Gram-negative bloodstream infection?*

*In what scenarios do you choose NOT to order follow-up blood cultures in patients with Gram-negative bloodstream infection?*

Patient responding/not responding to current therapy

Going to change/Not going to change management based on culture results

Data/no data to suggest benefit of follow up blood cultures

Patient considered/not considered high-risk

Patient does/does not have foreign device

Other

If responded “Always” – directed to this question:

Why do you always order follow-up blood cultures in patients with Gram-negative bloodstream infection?

Would change management based on culture results

Standard of practice in affiliated hospital

Data to suggest benefit of follow up blood cultures

Other

**Hypothetical Case Scenarios**

The following questions present fictional gram-negative bacteremia scenarios. For each scenario, imagine that you have been asked to provide an opinion about performing follow-up blood cultures. Where a clinical variable relevant to your choice is not described, please assume the average conditions seen in your practice.

For each scenario, we asked the respondent to select one of the following management selections:

*Which follow up would you recommend?*

*A) No follow up blood cultures (FUBC) needed*

*B) Yes, FUBC are needed*

**Case 1 (AIMS Score = 1)**

You are consulted on a 23-year-old female with *E. coli* bacteremia secondary to uncomplicated urinary tract infection. The patient has no relevant past medical history.  Initial blood cultures are positive. She is receiving Ceftriaxone for antibiotic therapy. She has no known history of antibiotic resistance. The patient initially demonstrated features of sepsis but has since stabilized.

**Case 2 (AIMS Score = 4)**

You are consulted on a 66-year-old female with *Pseudomonas aeruginosa* bacteremia. She is hospitalized for recurrent left leg stump osteomyelitis despite recent operative debridement. The patient’s medical history is significant for diabetes mellitus with peripheral vasculopathy, end stage renal disease on hemodialysis through a fistula, and amputation of the left leg. Previous tissue cultures show pan-susceptibility. Initial blood cultures are positive. She is receiving Ciprofloxacin for antibiotic therapy. The patient initially demonstrated features of sepsis but has since stabilized.

**Case 3 (AIMS Score = 0)**

You are consulted on a 70-year-old male with *Bacterioides fragilis* bacteremia secondary to right buttock cellulitis near medication injection site. The patient’s medical history is significant for poorly controlled diabetes mellitus, chronic kidney disease and alcohol abuse. Initial blood cultures are positive. He is receiving piperacillin-tazobactam for antibiotic therapy. The patient initially demonstrated features of sepsis but has since stabilized.

**Case 4 (AIMS Score = 1)**

You are consulted on a 57-year-old male with *Proteus mirabilis* bacteremia secondary to left-sided pyelonephritis. He has prior urinary tract infections with both susceptible and EBSL producing organisms. Initial blood cultures are positive. He was started on Ceftriaxone. The patient’s medical history is significant for recurrent renal stones. The patient initially demonstrated features of sepsis but has since stabilized.

**Case 5 (AIMS Score = 3)**

You are consulted on a 55-year-old female with *Morganella morganii* bacteremia secondary to ventricular peritoneal shunt infection. The patient’s past medical history is significant for left parietal lobe glioblastoma complicated by hydrocephalus. Initial blood cultures are positive. She is receiving meropenem for antibiotic therapy. The patient initially demonstrated features of sepsis but has since stabilized.

**Case 6 (AIMS Score = 1)**

You are consulted on a 29-year-old female with *Klebsiella pneumoniae* bacteremia secondary to perinephric abscess that is not amenable to drainage. The patient’s medical history is significant for active injection drug use and urinary tract infections. Initial blood cultures are positive. She was started on piperacillin-tazobactam. Her prior antibiotic resistance patterns are unknown. The patient initially demonstrated features of sepsis but has since stabilized.

**Case 7 (AIMS Score = 7)**

You are consulted on a 64-year-old male with *Serratia marcescens* bacteremia secondary to a pacemaker infection. The patient’s medical history is significant for end-stage related disease requiring hemodialysis, hypertension, cardiomyopathy, and left knee replacement 6 years ago. Initial blood cultures were positive. He is receiving Cefepime for antibiotic therapy. His line was removed 1 day ago. The patient initially demonstrated features of severe sepsis but has since stabilized.

**Case 8 (AIMS Score = 1)**

You are consulted on a 70-year-old male with *Stenotrophomonas maltophilia* bacteremia secondary to pneumonia. The patient’s past history is significant for end stage renal disease and he underwent kidney transplantation 10 days ago. Initial blood cultures were positive. He is receiving trimethoprim/sulfamethoxazole and levofloxacin for antibiotic therapy. His medications include tacrolimus and prednisone. He remains critically ill in the ICU after 48 h of antibiotics.

**Case 9 (AIMS Score = 2)**

You are consulted on a 65-year-old male with *Klebsiella pneumoniae* bacteremia secondary to cholecystectomy due recurrent cholecystitis. The patient’s medical history is significant for diabetes mellitus, chronic obstructive pulmonary disease, and myocardial infarction one year ago. The patient received empiric antibiotic coverage with piperacillin-tazobactam for 2 days but was later switched to meropenem after he developed a fever with persistent leukocytosis and sensitivities revealed an ESBL producing organism. The patient initially demonstrated features of sepsis but has since stabilized.

**Case 10 (AIMS Score = 3)**

You are consulted on a 55-year-old male with *Pseudomonas aeruginosa* bacteremia secondary to lumbar spondylitis with a small epidural abscess. The patient’s medical history is significant for Hodgkin’s lymphoma in remission for 1 year. There are no plans for surgical intervention. Initial blood cultures are positive. He is receiving meropenem for antibiotic therapy. Microbiological resistance profile demonstrates sensitivity to meropenem but he had a fever and elevated C-reactive protein 48 hours after starting antibiotics. He is hemodynamically stable.

**Supplemental Figures**

**Supplemental Figure S1.** Clinical practices and perspectives on bacteremia management (n=791). (A) Primary focus of practice among clinician respondents. (B) Self-reported monthly gram-negative bacteremia (GNB) caseload per respondent. (C) Key management actions in response to a positive blood culture for GNB, with recommendation to order blood cultures, selected action highlighted in blue. (D) Clinical metrics used to assess unfavorable response to treatment in patients with bacteremia. Responses reflect individual clinician practices and may exceed 100% due to multiple selections per respondent, as indicated (*).

**Table S1.** Clinician responses to management approaches to known diagnosis of bacteremia (n=791). Responses reflect individual clinician practices and may exceed 100% due to multiple selections per respondent.

**Table S2.** Clinician responses when asked which factors indicate unfavorable clinical response in patients with bacteremia (n=791). Responses reflect individual clinician practices and may exceed 100% due to multiple selections per respondent.

**Table S3.** Clinician responses to management approaches to known diagnosis of bacteremia as stratified by geographic region (n=791). “Other” includes clinicians from continents other than North America. Responses reflect individual clinician practices and may exceed 100% due to multiple selections per respondent.

**Table S4.** Clinician responses when asked which factors indicate unfavorable clinical response in patients with bacteremia (n=791). “Other” includes clinicians from continents other than North America. Responses reflect individual clinician practices and may exceed 100% due to multiple selections per respondent.

**Table S5.** Survey responses indicating whether follow-up blood cultures (FUBCs) would be obtained for 10 hypothetical cases of gram-negative bacteremia, stratified by geographic region (n=691). Percentages represent clinicians recommending FUBCs. “Other” includes clinicians from continents other than North America.

**Table S6.** Clinician responses to management approaches to known diagnosis of bacteremia as stratified by continent (n=791). Responses reflect individual clinician practices and may exceed 100% due to multiple selections per respondent. Inferential statistics for this comparison are not provided given that two of the continents have fewer than 10 respondents.

**Table S7.** Clinician responses when asked which factors indicate unfavorable clinical response in patients with bacteremia stratified by continent (n=791). Responses reflect individual clinician practices and may exceed 100% due to multiple selections per respondent. Inferential statistics for this comparison are not provided given that two of the continents have fewer than 10 respondents.

**Table S8.** Survey responses indicating whether follow-up blood cultures (FUBCs) would be obtained for 10 hypothetical cases of gram-negative bacteremia, stratified by continent (n=691). Percentages represent clinicians recommending FUBCs. Inferential statistics for this comparison are not provided given that two of the continents have fewer than 10 respondents.

**Table S9.** Management recommendations for a known diagnosis of bacteremia stratified by clinicians’ years since completion of training (n=791). Responses reflect individual clinician practices and may exceed 100% due to multiple selections per respondent.

**Table S10.** Clinician responses when asked which factors indicate unfavorable clinical response in patients with bacteremia, stratified by clinicians’ years since completion of training (n=791). Responses reflect individual clinician practices and may exceed 100% due to multiple selections per respondent.

**Table S11.** Survey responses indicating whether follow-up blood cultures (FUBCs) would be obtained for 10 hypothetical cases of gram-negative bacteremia, stratified by respondents’ years since completion of training (n=691). Percentages represent respondents recommending FUBCs.

**Table S12.** Management recommendations for a known diagnosis of bacteremia stratified by Infectious Diseases (ID) versus non-ID specialists (n=791). Responses reflect individual clinician practices and may exceed 100% due to multiple selections per respondent.

**Table S13.** Respondent responses when asked which factors indicate unfavorable clinical response in patients with bacteremia, stratified by Infectious Diseases (ID) versus non-ID specialists (n=791). Responses reflect individual clinician practices and may exceed 100% due to multiple selections per respondent.

**Table S14.** Survey responses indicating whether follow-up blood cultures (FUBCs) would be obtained for 10 hypothetical cases of gram-negative bacteremia, stratified by Infectious Diseases (ID) versus non-ID specialists (n=691). Percentages represent respondents recommending FUBCs.

**Table S15.** Management recommendations for a known diagnosis of bacteremia stratified by hospital category (n=791). Responses reflect individual clinician practices and may exceed 100% due to multiple selections per respondent.

**Table S16.** Clinician responses when asked which factors indicate unfavorable clinical response in patients with bacteremia, stratified by hospital category (n=791). Responses reflect individual clinician practices and may exceed 100% due to multiple selections per respondent.

**Table S17.** Survey responses indicating whether follow-up blood cultures (FUBCs) would be obtained for 10 hypothetical cases of gram-negative bacteremia, stratified by hospital category (n=691). Percentages represent respondents recommending FUBCs.

**Table S18.** Survey responses (n=691) indicating whether follow-up blood cultures (FUBCs) would be obtained for 10 hypothetical cases of gram-negative bacteremia, stratified by how respondents answered the survey question: “In your clinical practice, how often do you order follow-up blood cultures in patients with gram-negative bloodstream infection?” Response selections included: 1) Never; 2) Depends on the scenario; 3) Always. Percentages represent respondents recommending FUBCs.

**References**

1.	Maskarinec SA, Park LP, Ruffin F, Turner NA, Patel N, Eichenberger EM, van Duin D, Lodise T, Fowler VG, Jr., Thaden JT. Positive follow-up blood cultures identify high mortality risk among patients with Gram-negative bacteraemia. Clin Microbiol Infect. 2020;26(7):904-10. PubMed PMID: 32114010.

2.	Peleg AY, Hooper DC. Hospital-acquired infections due to gram-negative bacteria. N Engl J Med. 2010;362(19):1804-13. PubMed PMID: 20463340.

3.	Keaton A, Fike L, Spicer K, Kallen A, Perkins K. Healthcare-associated *Stenotrophomonas maltophilia* infections in the United States, 2018–2022. Antimicrob Steward Healthc Epidemiol. 2023;3(Suppl 2):s89.

4.	Mahlen SD. *Serratia* infections: from military experiments to current practice. Clin Microbiol Rev. 2011;24(4):755-91. PubMed PMID: 21976608.

5.	Eichenberger EM, Ruffin F, Dagher M, Lerebours R, Jung SH, Sharma-Kuinkel B, Macintyre AN, Thaden JT, Sinclair M, Hale L, Kohler C, Palmer SM, Alexander BD, Fowler VG, Jr., Maskarinec SA. Bacteremia in solid organ transplant recipients as compared to immunocompetent patients: Acute phase cytokines and outcomes in a prospective, matched cohort study. Am J Transplant. 2021;21(6):2113-22. PubMed PMID: 33131212.
